# Structured graphene metamaterial selective absorbers for high efficiency and omnidirectional solar thermal energy conversion

**DOI:** 10.1038/s41467-020-15116-z

**Published:** 2020-03-13

**Authors:** Keng-Te Lin, Han Lin, Tieshan Yang, Baohua Jia

**Affiliations:** 1 Centre for Translational Atomaterials, Faculty of Science, Engineering and Technology, Swinburne University of Technology, P.O. Box 218, Hawthorn, VIC 3122 Australia; 2 The Australian Research Council (ARC) Industrial Transformation Training Centre in Surface Engineering for Advanced Materials (SEAM), PO Box 218, Hawthorn, VIC 3122 Australia

**Keywords:** Devices for energy harvesting, Metamaterials, Optical properties and devices

## Abstract

An ideal solar-thermal absorber requires efficient selective absorption with a tunable bandwidth, excellent thermal conductivity and stability, and a simple structure for effective solar thermal energy conversion. Despite various solar absorbers having been demonstrated, these conditions are challenging to achieve simultaneously using conventional materials and structures. Here, we propose and demonstrate three-dimensional structured graphene metamaterial (SGM) that takes advantages of wavelength selectivity from metallic trench-like structures and broadband dispersionless nature and excellent thermal conductivity from the ultrathin graphene metamaterial film. The SGM absorbers exhibit superior solar selective and omnidirectional absorption, flexible tunability of wavelength selective absorption, excellent photothermal performance, and high thermal stability. Impressive solar-to-thermal conversion efficiency of 90.1% and solar-to-vapor efficiency of 96.2% have been achieved. These superior properties of the SGM absorber suggest it has a great potential for practical applications of solar thermal energy harvesting and manipulation.

## Abstract (web summary)

Here, the authors demonstrate a selective solar thermal absorber with wavelength selectivity, arising from metallic trench-like structures, using broadband dispersionless ultrathin graphene metamaterial film, with excellent thermal conductivity.

## Introduction

The sun as a life enabler provides the earth with gigantic energy in the form of light and heat. As energy scarcity becomes one of the most serious global challenges, humanity devotes considerable efforts to search technological solutions for solar energy harvesting. Solar energy harvesting is usually performed in one of the three means: the photovoltaic approach^1–3^, which converts photon energy directly to electricity by photovoltaic devices; the photochemical approach^4,5^, which converts solar energy to storable chemical fuels, such as hydrogen; or the photothermal approach^6–14^, in which photons are converted into thermal energy by solar-thermal absorbers. The major advantages of the last strategy are the exploitation of a broader bandwidth within the solar spectrum and therefore it enables higher conversion efficiencies and it is environmentally friendly with almost the smallest carbon footprint among all the energy harvesting approaches (Supplementary Fig. 1 and Supplementary Note 1).

In a photothermal system, the performance of the solar-thermal absorbers holds the key and can be quantitatively characterized by the overall efficiency of solar-thermal power generation system, which can be expressed using Eq. (1)^6^:

1 $$\eta = \eta _{{\mathrm{solar}} \;{\mathrm{thermal}}}\left( {1 - \frac{{T_0}}{{T_{\mathrm{A}}}}} \right),$$

where *ƞ* is the overall efficiency of the solar-thermal power generation system, *ƞ*_solar thermal_ is the solar-to-thermal conversion efficiency, *T*_0_ is the ambient temperature, and *T*_A_ is the working temperature of solar-thermal absorbers. Among these parameters, solar-to-thermal conversion efficiency based on energy balance equation can be further expressed using Eq. (2)^6,7^:

2 $$\eta _{{\mathrm{solar}}\;{\mathrm{thermal}}} = E_\upalpha - E_{\mathrm{R}} = \frac{{C \times {\int} {{\mathrm{d}}\lambda \;\alpha \left( \lambda \right)E_{\mathrm{solar}}\left( \lambda \right)} - {\int} {{\mathrm{d}}\lambda \;\alpha \left( \lambda \right)E_{\mathrm{B}}\left( {\lambda ,T_{\mathrm{A}}} \right)} }}{{C \times {\int} {{\mathrm{d}}\lambda \;E_{\mathrm{solar}}\left( \lambda \right)} }},$$

where *E*_α_ is the total solar absorbance, *E*_R_ is the thermal radiation loss, *E*_solar_ is the spectral solar irradiation, *E*_B_ (*λ*,*T*_A_) is the black-body radiation at temperature *T*_A_, and *C* is the concentration factor that is usually on the order of 1 to 1000. For fixed temperatures of *T*_0_ and *T*_A_, a higher solar-to-thermal conversion efficiency leads to a higher overall efficiency. To reach the highest solar-to-thermal conversion efficiency, it is essential to maximize the total solar absorbance (*E*_α_), while minimizing the thermal radiation loss (*E*_R_). In an ideal case, there exists a step function-like spectral selection mechanism for the solar-thermal absorbers at a specific temperature (Supplementary Fig. 2). Such a step function has a 100% absorption overlapping as broad as possible with the solar spectrum and a 0% emission for the infrared (IR) range starting at the cut-off wavelengths, which is at the intersection of the solar spectrum (*C* × *E*_solar_) and the emission spectrum (*E*_B_)^6^ (Supplementary Fig. 2). Therefore, from the ideal solar-thermal absorber design point of view, selective and almost total absorption across the entire solar spectrum, flexible tunability of the cut-off wavelength, and minimized excessive energy dissipation by thermal radiation in the near-IR (NIR) to mid-IR range are the essential factors.

In general, solar-selective absorbers can be divided into three distinct types based on their thermal modulation strategies: multi-layer thin film coating^15–17^, plasmonic nanostructures^7–11,18–22^, and carbon-based materials^12–14,23–25^. Although many demonstrations of solar-thermal absorbers have been realized by different strategies (Supplementary Note 2), the challenges remain on the limitations of naturally available materials that can simultaneously satisfy the stringent requirements of the practical solar-thermal absorbers for high thermal conductivity, low thermal radiation loss (i.e., low emissivity over several tens of micrometers of wavelength in the IR regime) and wavelength-selective mechanism from a simple strategy.

In this paper, we propose and demonstrate a new concept for developing a selective solar-thermal absorber from a three-dimensional (3D) structured graphene metamaterial (SGM) on metal substrates. The concept of 3D SGM design combines the advantages of wavelength selectivity from the trench-like structures and the broadband dispersionless nature and high thermal conductivity from the ultrathin graphene metamaterials^26–30^ (Supplementary Note 3 and Supplementary Table 1). Therefore, this concept is fundamentally different from those of carbon-based absorbers^12–14,23–25^ and plasmonic nanostructures^6–11^, where selectivity and broadband are challenging to achieve simultaneously. Previously, a 90-nm-thick graphene grating metamaterial with ~85% absorptivity of unpolarized light covering almost the entire solar spectrum was demonstrated^29^. However, the ultrabroadband absorption is incapable of designing a selective solar absorber due to the challenge in limiting the absorption in the desired cut-off wavelength range. Herein, we demonstrate that by conformally coating a 30-nm-thick graphene metamaterial on a 3D trench-like structure to form the SGM, the exact absorption band for efficient solar-selective absorption can be precisely controlled. Almost all the incident light can be absorbed within the 30 nm ultrathin graphene metamaterial and the SGM retains wavelength tunability and selectivity. Moreover, the SGM absorbers possess efficient and omnidirectional solar absorption capability, high solar-to-thermal conversion efficiency, superior photothermal performance, and high thermal stability. Therefore, the 3D SGMs feature not only the passive modulation of photothermal behavior but also active functions to transport the photo-generated heat and protect the underlying structured metal due to the excellent thermal conductivity and impermeability of the graphene metamaterials, suggesting promising practical applications in solar-thermal energy harvesting.

## Results

### Design of 3D SGM absorber

Due to its dispersionless nature^29^, the graphene metamaterial possesses broadband absorption from 0.28 to 25 µm even only at 30 nm in thickness (Supplementary Fig. 3). When the thin graphene metamaterial, which consists of alternating graphene and dielectric layers^29,30^, is deposited on metallic substrates, for example, copper, the absorbance of the hybrid materials in the visible regime is significantly enhanced and at some wavelength it reaches almost 100%. In the meantime, the absorbance in the IR regime is dramatically suppressed to almost zero due to the strong interference phenomena^31^ (Supplementary Fig. 3). Nevertheless, the total solar absorbance (*E*_α_) is not close to unity and there is no tuning mechanism presenting in this system allowing flexibly tunable absorption bandwidth to meet the requirements of efficient selective solar-thermal absorbers.

**Fig. 1:**
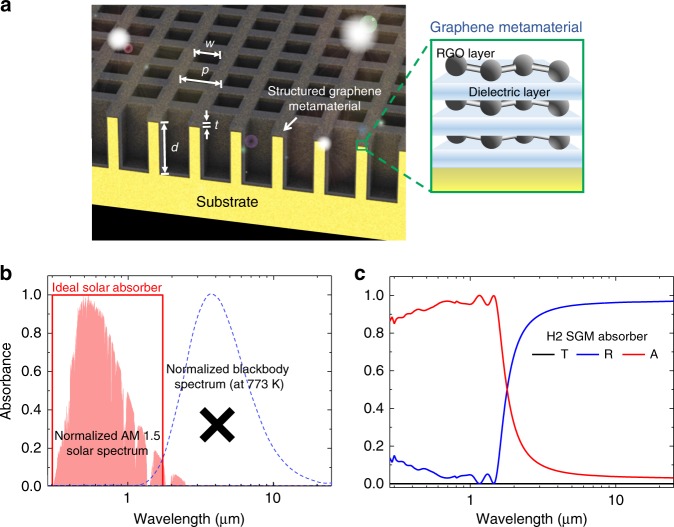
SGM solar-selective absorber. **a** Schematic representation of the 3D SGM absorber, characterized by the structural parameters: the depth (*d*) of trenches, the thickness (*t*) of the graphene metamaterial layer, the width (*w*) of the hole, and the period (*p*) of the structure. Inset shows the structure of the graphene metamaterial. **b** Absorbance spectrum of ideal solar absorber. Ideal solar absorber is able to efficiently absorb (near-unity) sunlight (shaded red) and minimize energy dissipation through thermal radiation (blue dash line). X indicates the blackbody thermal emission is inhibited. **c** Simulated spectral reflectance (*R*), transmittance (*T*), and absorbance (*A*) for the SGM absorber featuring a hole width of 0.59 µm, a period of 0.8 µm, a depth of trenches of 1 µm, and a graphene metamaterial film with a thickness of 30 nm.

To enable the flexible tuning mechanism, we propose a concept of 3D SGM for the development of solar-thermal absorbers. Figure 1a illustrates the schematic design of the 3D SGM solar-thermal absorber, which comprises a graphene metamaterial layer we recently developed^29^ conformally coated on the 3D trench-like metal substrate and patterned in a matrix arrangement. The nanostructure is designed in such a way that it should have the *C4* rotational symmetry to realize polarization-independent absorption. The key structural parameters of the 3D SGM include the depth (*d*) of trenches, the thickness (*t*) of graphene metamaterial layer, the width (*w*) of the holes, the period (*p*) of the structure, and the species of the metal. These parameters can be used to precisely manipulate the photothermal properties of the solar-selective absorber, which are close to those of ideal solar-thermal absorber as illustrated in Fig. 1b.

In the design principle, the coupled light in the nanostructure will form a cavity resonance as a standing wave inside the trench. Therefore, the width should be chosen as $w = \frac{{\lambda _{{\mathrm{cut}}{\mbox{-}}{\mathrm{off}}}}}{{2n_{\mathrm{eff}}}} - 2t$, where *λ*_cut-off_ is the cut-off wavelength and *n*_eff_ is the refractive index of the materials inside the trenches. In this case, it is the effective refractive index of the graphene metamaterial and air. Although the wavelength used here is the cut-off wavelength of the absorption band, the light with shorter wavelengths can still propagate into the cavity and then be absorbed by the side wall of the SGM. In this way, broadband absorption can be achieved. On the other hand, *w* decides the cut-off wavelength of the solar-selective absorber, for details please see Supplementary Fig. 4a. The mode at the longer wavelength cannot be supported by the structure, and thereby, it can be effectively cut off. To effectively eliminate reflection of the SGM absorber in ultraviolet (UV) to NIR regime, the period should be chosen as *p* − *w* ≪ *λ*. In addition, the light is absorbed when it propagates along the side wall of the trench due to the conformal coating of the graphene metamaterial on the side wall. Therefore, the minimum depth required depends on the effective extinction coefficient of the graphene metamaterial (*k*_gm_) at the cut-off wavelength, in the form of $d = \frac{{\lambda _{{\mathrm{cut}}{\mbox{-}}{\mathrm{off}}}}}{{4\pi k_{\mathrm{gm}}}}$. The synergetic effect of these structural parameters allows the design of high-performance selective absorber in the wavelength range according to desired application requirements. In this study, we take the solar-selective absorber to demonstrate the validity of the design principle and selective absorption performance of the SGM absorber.

To analyze the solar-selective absorption capability of our 3D SGM absorber, we used 3D finite-difference time-domain (3D-FDTD) method to map out and optimize the optical behaviors of the SGM absorbers and selected three sets of parameters and displayed in Supplementary Table 2 as H1, H2, and H3. As the thickness of the copper substrate is >100 µm, the transmittance is *T* = 0. Therefore, the absorbance (*A*) can be calculated as *A* = 1 − *R*, where *R* is the reflectance. As shown in Fig. 1c, with a graphene metamaterial layer of 30 nm, the reflectance of the H2 SGM absorber is sufficiently low in a wide wavelength range from 0.28 to 1.6 µm and increases significantly in the IR regime (from 1.6 to 2.5 µm) while the transmittance is zero. Therefore, the proposed SGM absorber could achieve solar-selective absorption, with the average absorption of 95% in the solar spectral range from 0.28 to 1.6 µm and the emissivity of as low as 3.9% in the IR range. Interestingly, the optical properties of SGM would barely be affected by the substrate materials, suggesting that the SGM could be fabricated with low-cost metals, for example, aluminum (Supplementary Fig. 4b), making it practically attractive.

The absorbance of the 3D SGM with respect to the incident wavelength can be tuned readily by varying the hole width, period, and depth of trenches while fixing the thickness of the graphene metamaterial layer. As displayed in Supplementary Fig. 5, upon simultaneously enlarging the hole width, period, and depth of trenches of the SGM absorber, the absorption bandwidth enlarges obviously from 1.2 (0.28–1.48 µm) to 1.77 µm (0.28–2.05 µm) without losing the step function selectivity. It indicates that the proposed SGM absorber has an excellent wavelength tunability for selective solar absorption, which could be used in a wide range of working temperatures.

**Fig. 2:**
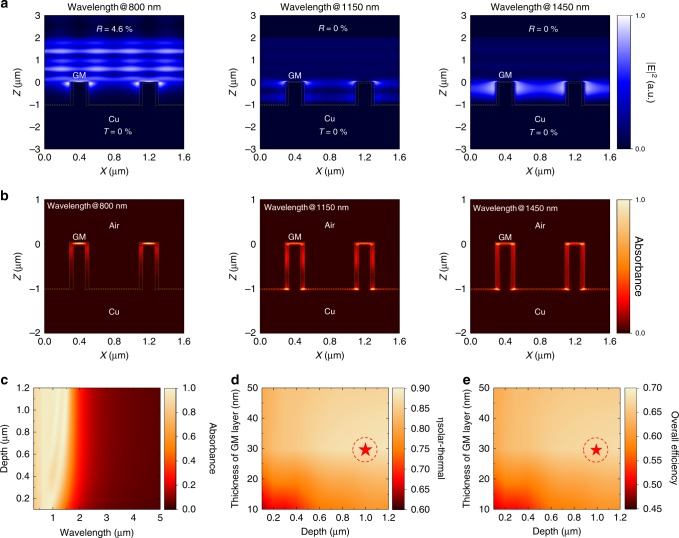
Optical properties and efficiencies of SGM absorbers. **a**, **b** Simulated **a** electric field intensity distributions and **b** absorbance map of incident light at various wavelengths (800, 1150, and 1450 nm) passing through the H2 SGM absorber. **c** Absorption spectra for SGM absorber featuring *w* = 0.59 µm, *p* = 0.8 µm, and *t* = 30 nm as a function of depth of trenches and wavelength. **d**, **e** Calculated **d** solar-to-thermal efficiencies and **e** overall efficiencies of the SGM absorber featuring *w* = 0.59 µm and *p* = 0.8 µm as a function of thickness of the graphene metamaterial (GM) layer and depth of trenches at *T*_0_ = 298 K, *T*_A_ = 1273 K, and *C* = 1000. Circled red star indicates the optimized structural parameters (*d *= 1 µm and *t *= 30 nm) of SGM absorber.

The absorption mechanism of the 3D SGM absorber is further revealed by analyzing the electric field distributions at distinct wavelengths. As displayed in Fig. 2a, the incident light with different wavelengths through the H2 SGM absorber with a 30-nm graphene metamaterial layer is simulated and the electric field distributions are recorded in stable states. We found that the 3D SGM absorber provides a strong electric field within the near field around the air–graphene metamaterial interface when the wavelengths are at 800, 1150, and 1450 nm, respectively. Thus, this absorber exhibits both low reflectance (*R* = 4.6%/0%/0% at 800 nm/1150 nm/1450 nm, respectively) and low transmittance (*T* = 0%/0%/0% at 800 nm/1150 nm/1450 nm, respectively) over a broad wavelength regime simultaneously. We attribute this result to the structure featuring both surface plasmon resonance and cavity effects to trap the incident light around the graphene metamaterial layer in the near field. The large effective area of the graphene metamaterial sidewalls within the high electric field intensity regime leads to most of the light being absorbed by the ultrathin graphene metamaterial layer, as displayed in Fig. 2b, and subsequently be converted to thermal energy. In addition, the dimension of the hole width of the H2 SGM absorber relative to the incident wavelengths in IR regime is too small that incident light can hardly propagate into the cavities, and thereby, this absorber performs high reflectance and low emissivity over the IR regime (Supplementary Fig. 6). Therefore, the photo-generated heat in the graphene metamaterial layer on SGM absorber can be readily conducted through this continuous metamaterial film for further usage since the graphene metamaterials feature excellent thermal conductivity, while hardly dispersed by thermal radiation.

Although it is possible to directly derive near-optimal structural parameters based on the design principle, further optimization has been performed to find out the optimal design of the 3D SGM absorber to achieve the best solar-thermal conversion performance. As displayed in Fig. 2c, the spectral absorbance of the SGM absorber with a *w* = 0.59 µm, *p* = 0.8 µm, and *t* *=* 30 nm was simulated when the depth of trenches varied from 0.1 to 1.2 µm. We found that the SGM could maintain solar-selective absorption capability at distinct depth of trenches, and the absorption bandwidth is enlarged gradually with increasing the depth of trenches. Furthermore, the spectral absorbance keeps almost the same when the depth of trenches is >1 µm. These phenomena also exist for the SGM absorbers with distinct thicknesses of graphene metamaterial layer (Supplementary Fig. 7).

The solar-to-thermal efficiencies and the overall efficiencies of the 3D SGM absorbers are calculated based on the spectral absorbance of SGMs with different structural parameters (Fig. 2c and Supplementary Fig. 7). As shown in Fig. 2d, e, ultra-high solar-to-thermal efficiency of 88% and overall efficiency of 68% at *T*_0_ = 298 K, *T*_A_ = 1273 K, and *C* = 1000 are achieved when the SGM absorber has a 30-nm graphene metamaterial layer and 1-µm depth of trenches. It is worth noting that the trench-like nanostructures can further enhance the interaction length of the metamaterial and light, thus much thinner film (30 nm) with larger absorbance can be achieved compared to the grating graphene metamaterial (90 nm) in our previous work^29^.

**Fig. 3:**
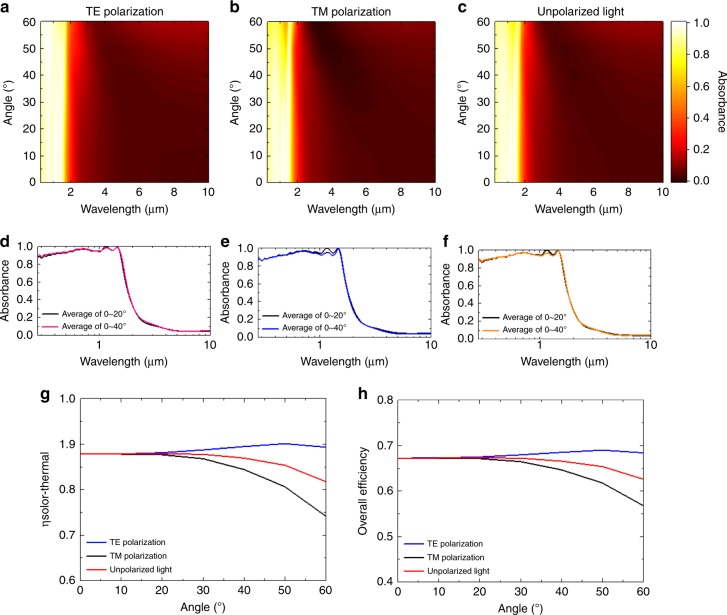
Omnidirectional solar-thermal energy conversion capability of SGM absorber. **a**–**c** Angle-dependent absorption spectra of H2 SGM absorber with 30-nm graphene metamaterial film under **a** TE-polarized, **b** TM-polarized, and **c** unpolarized light, respectively. **d**–**f** Averaged absorbance over incident angles from 0° to 20° and 0° to 40° of **d** TE-polarized, **e** TM-polarized, and **f** unpolarized light. **g**, **h** The calculated **g** solar-to-thermal efficiencies and **h** overall efficiencies of H2 SGM absorber with a 30-nm graphene metamaterial film under TE-polarized, TM-polarized, and unpolarized light at various angles of incidence and the working situation of *T*_0_ = 298 K, *T*_A_ = 1273 K, and *C* = 1000.

Omnidirectional absorption is one of the important characteristics for a solar-selective absorber to efficiently absorb the sunlight from a large angle range. Here, we investigate the angular dependence of the absorption spectrum and solar-to-thermal energy conversion capability of the 3D SGM absorber. Figure 3a–c display the mapping of the absorption spectra simulated at different angles of incidence for the H2 SGM absorber with a 30-nm graphene metamaterial under transverse electric (TE) polarized, transverse magnetic (TM) polarized, and unpolarized light, respectively. The averaged absorbance spectra are calculated based on the simulated spectral absorption over incident angles of 0° to 20° and 0° to 40° as displayed in Fig. 3d–f. The solar-selective absorption of the SGM absorber maintains almost the same for a wide range of incident angles from 0° to 50° under TE-polarized (Fig. 3a, d), TM-polarized (Fig. 3b, e), and unpolarized light (Fig. 3c, f). The spectral absorption of the SGM absorber under TE-polarized light maintain well even when the incident angle increases up to 60°, while they varied under TM polarization, especially when the angle of incidence is large. We attribute this phenomenon to the decreased intensities of the electric field, which is perpendicular to the trench-like metallic structures, under oblique incidence of the TM polarization. Therefore, the light-SGM absorber interactions decrease and, accordingly, the absorption also decreases. Based on the simulated absorption spectra we calculate the corresponding *ƞ*_solar thermal_ and *ƞ* at *T*_0_ = 298 K, *T*_A_ = 1273 K, and *C* = 1000, as displayed in Fig. 3g, h, respectively. The SGM absorber performs high *ƞ*_solar thermal_ and *ƞ* of 90.1% and 68.9%, respectively, under TE-polarized light with an incident angle of 50°. The *ƞ*_solar thermal_ and *ƞ* under TM polarization are maintained higher than 80.6% and 61.7%, respectively, for incident angles up to 50°. When contemplating the light collection capability under unpolarized light, which is the most practical circumstances in the applications of solar-thermal energy conversion, the *ƞ*_solar thermal_ and *ƞ* remain nearly constants of >86.9% and 66.6%, respectively, for the range of incident angle between 0° and 40°. They are decreased slightly but are still >85.3% and 65.3%, respectively, for incident angles up to 50° under unpolarized light. In addition, the SGM absorber exhibits excellent performance at various working situations (e.g., *T*_0_ = 298 K, *T*_A_ = 473 K, and *C* = 1 and 10) as shown in Supplementary Fig. 8. As a result, the SGM strategy features omnidirectional absorption and high solar-thermal energy conversion capabilities under unpolarized light, suggesting that the proposed SGM absorber has promising potentials for use in practical applications of solar-thermal energy conversion.

### Fabrication of SGM absorber and experimental results

**Fig. 4:**
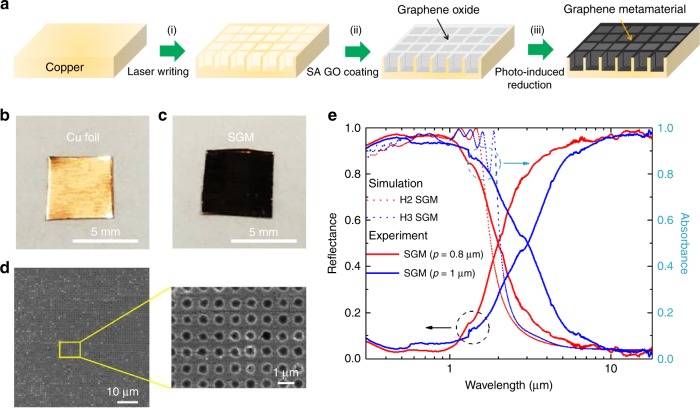
Fabrication and characterization of SGM absorbers. **a** Schematic representations of steps in fabrication processes for preparing 3D SGM absorbers. **b**, **c** Photographs of **b** Cu foil and **c** SGM absorber, each having sample area of ~25 mm^2^. **d** Top-view SEM images of the SGM absorber at low and high magnification. **e** Measured reflectance and absorbance spectra of SGM absorbers having various hole widths (*w*) and periods (*p*): that is, *w*/*p* = 0.52/0.8 μm (red line) and *w*/*p* = 0.57/1 μm (blue line). The red dashed line and the blue dashed line are the simulated absorbance spectra of the H2 and H3 SGM absorbers, respectively.

To verify the concept of 3D SGM design, we used standard laser nanofabrication^32^, self-assembly graphene oxide (GO) coating^30^, and photo-induced reduction technologies to fabricate SGM absorbers (see Methods). The critical fabrication steps are displayed in Fig. 4a. The trench-like structures were first fabricated on the Cu substrate using direct laser printing setup with a femtosecond laser^33^ [step (i)]. Here, the SGM absorbers featuring different dimensions of hole width, period, and depth of trenches can be prepared readily in this single step of fabrication. After patterning, the structured Cu substrate was cleaned using deionized (DI) water to remove any ablated Cu particles. Then, a 40-nm GO metamaterial film was deposited by water-based self-assembly process [step (ii)]. The GO film conformally coated the patterned substrate to form 3D GO cavities through the sidewalls of the trenches. Finally, the GO-coated sample was illuminated with a mercury lamp to achieve ~30-nm graphene metamaterial^30^ through photoreduction, providing a solar-thermal absorber possessing 3D SGM [step (iii)]. Figure 4b, c, and Supplementary Fig. 9a reveal the photograph of the Cu foil, SGM absorber, and flat Cu foil coated with graphene metamaterial (GM@Cu foil), respectively. It is obvious that the original brass foil (Fig. 4b) became black after fabricating the SGM on it (Fig. 4c), which directly indicates the efficient and broadband absorption of the SGM absorber. Figure 4d and Supplementary Fig. 9b display the top-view scanning electron microscopic (SEM) images of the SGM absorbers. Herein, we prepared SGM absorbers featuring different hole widths and periods in a matrix symmetry on Cu substrates, which we fabricated with hole widths/periods of 0.6/0.8 µm (Supplementary Fig. 9b) and 0.62/1 µm (Fig. 4d), respectively. As displayed in Fig. 4d and Supplementary Fig. 9b, we can fabricate uniform SGM on the Cu substrate in a large area with the simple laser writing method.

The optical properties of the flat GM@Cu foil (Supplementary Fig. 10) and SGM absorbers under unpolarized lights were characterized with UV–visible–NIR spectrophotometer and Fourier transform IR (FTIR) spectrometer. When the Cu foil is coated with a 30-nm graphene metamaterial layer, it absorbed strongly in the UV and visible regime from 0.28 to 0.78 µm, but poorly in the NIR regime from 0.8 to 1.5 µm (Supplementary Fig. 10). Thus, the sunlight could not be efficiently collected by the flat GM@Cu foil. In contrast, the SGM absorber achieves solar-selective absorption and performs high absorbance (>80%) over broadband wavelengths, including UV, visible, and NIR regime, while keeping low absorbance (<5%) in the IR regime, as designed. Furthermore, the measured reflectance and absorbance spectra (Fig. 4e) indicate that varying the dimensions of the SGM absorber can effectively manipulate the bandwidth of reflectance and absorbance. As displayed in Fig. 4e, the average reflectance and absorbance of the SGM absorber with *w* = 0.6 µm and *p* = 0.8 µm reached ~8.7% and 91.3%, respectively, in the wavelength range from 0.28 to 1.6 µm. The bandwidth of absorbance higher than 80% of this structure is ~1.2 µm (from 0.28 to 1.48 µm). When the hole width and period of the SGM absorbers are increased to 0.62 and 1 µm, respectively, the averaged reflectance and absorbance for the SGM absorbers were ~9.7% and 90.3%, respectively, in the wavelength range from 0.28 to 1.8 µm. In this case, the bandwidth of absorbance >80% is ~1.49 µm (from 0.28 to 1.77 µm). Here, the simulated absorbance spectrum shows a reasonably good agreement with the experimental measurement demonstrating the effectiveness of the method. In particular, when the widths and periodicities of the structures are enlarged, the absorbance bandwidths of SGM absorbers are extended in both experiment and simulation. The measured absorbance spectra of these samples were slightly different from the simulated results, in particular for the SGM absorbers with large hole width and periodicity, presumably because the roughness of the sidewalls and the imperfect shapes of holes and trenches in the fabricated SGM absorbers, which are slightly differed from those in the simulations (Supplementary Fig. 11). The reflectance and absorbance spectra and the sharpness of the spectrum cut-off may be further improved through more optimized control of the fabrication process. However, on the other hand, the high-quality absorbance spectra also indicate the structural robustness and effectiveness of the proposed SGM absorber.

**Fig. 5:**
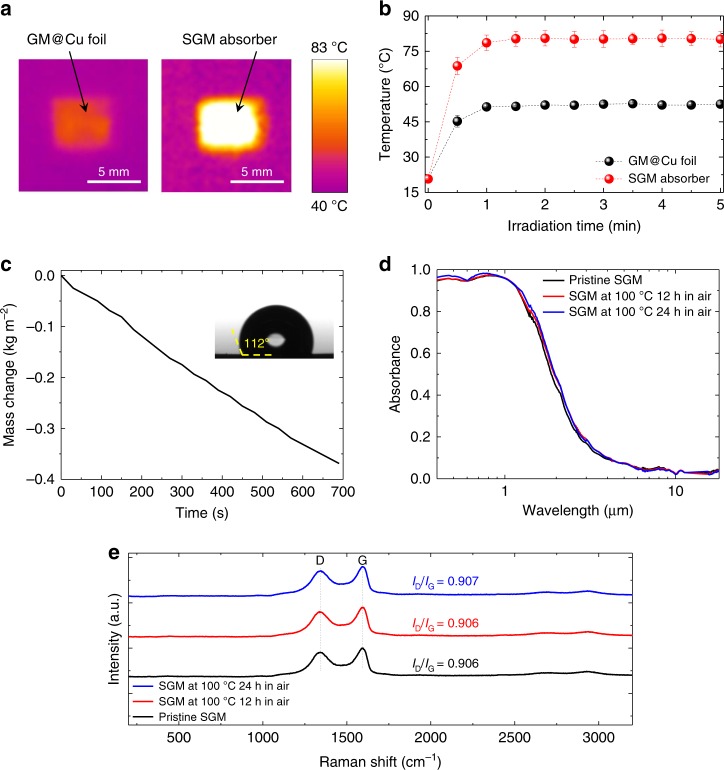
Photothermal application and thermal stability of SGM absorbers. **a** Thermal images of the flat GM@Cu foil and SGM absorber under sunlight illumination in an open environment. **b** Temperatures of the flat GM@Cu foil (black) and SGM absorber (red) after various periods of illumination. Error bars on the *Y*-axis show standard deviations calculated from the measured temperatures. **c** Mass change of water with SGM absorber as function of time under one sun illumination. Inset: surface wetting property of SGM absorber. **d**, **e** Measured **d** absorbance spectra and **e** Raman spectra of SGM absorbers before, after 12 h, and after 24 h of heating at 100 °C in air.

Figure 5a, b present the photothermal performance of the SGM absorber and graphene metamaterial on Cu foil under the solar simulator illumination in an open environment (see Methods). While illuminating the samples, we used a thermal imaging camera to record the surface temperature of the samples. During the first minute, the temperature of each sample increased dramatically, thereafter saturated gradually (Fig. 5b). As displayed in Fig. 5b, the temperature of graphene metamaterial on Cu foil sample increased from the room temperature (20 °C) to ~52 °C; in contrast, the temperature of SGM absorber increased significantly to ~80 °C (Fig. 5a). Therefore, the SGM absorber performed an excellent photothermal characteristics, presumably because a great amount of sunlight was absorbed by the ultrathin graphene metamaterial layer on the SGM (Fig. 2b); thereafter, the photo-generated heat could be transported rapidly to the whole SGM absorber leading to a high overall temperature in a short period of time.

The effectiveness of the SGM absorber is further demonstrated by evaluating its solar steam generation behavior. Figure 5c displays the time-dependent mass change of water on the SGM absorber under one sun illumination (see Methods). The average evaporation rate (m*) under one sun illumination is measured to be 1.5 kg m^−2^  h^−1^. Accordingly, the solar-to-vapor efficiency (*ƞ*_solar-to-vapor_) can be calculated based on the Eq. (3)^10^:

3 $$\eta _{{\mathrm{solar}}{\mbox{-}}{\mathrm{to}}{\mbox{-}}{\mathrm{vapor}}} = \frac{{m^ \ast h_{\mathrm{LV}}}}{{{\mathrm{CP}}_{\mathrm{sun}}}},$$

where *h*_LV_ is the latent heat of vaporization (i.e., ≈2308 J g^−1^ at 80 °C^34^), and *P*_sun_ is the power density of one sun (i.e., ≈1000 W m^−2^). The calculated *ƞ*_solar-to-vapor_ according to the experimentally measured average evaporation rate is 96.2%, which is higher than most of previously reported values^10–14^ due to the efficient photothermal conversion and high thermal conductivity and structural continuity of the SGM, which can effectively pass the converted thermal energy to the water layer. In addition, due to the surface property of graphene^35,36^ and the presence of trench-like nanostructures, the SGM absorber exhibits a hydrophobic property (inset of Fig. 5c), which helps to further improve the water evaporation rate^37^.

For practical solar-thermal energy conversion applications, an SGM absorber requires to maintain its performance under working situations for an extended period of time. Therefore, it needs to be thermally stable. To investigate the thermal stability of the SGM absorbers, we measured the absorbance spectra of the absorbers with and without thermal treatments at a temperature of 100 °C in air for 24 h. We then, based on these measured spectra, calculated *E*_α_, *E*_R_, and *ƞ*_solar thermal_ at working situations of *T*_A_ = 373 K and *C* = 1 and 10. As displayed in Fig. 5d, the absorption in the short wavelength regime and absorption bandwidth of the SGM absorbers increased slightly after thermal treatments. We attribute this phenomenon to the slight increments of the reduction degree of GO at 100 °C. Nevertheless, the absorptions of SGM absorbers with and without thermal treatments retain almost the same in the IR regime. Therefore, as indicated in Supplementary Table 3, *E*_α_ increased and *E*_R_ decreased slightly with prolonged heating at 100 °C and *C* = 1. The performance of SGM absorbers can maintain the stability within 24 h in air at 100 °C, with *E*_α_ slightly increased from 90.5% to 91.9% and *E*_R_ slightly decreased from 4.1% to 3.8%. Accordingly, *ƞ*_solar thermal_ essentially increased from 86.3% to 88.1% and from 90.1% to 91.5% at concentration factors of 1 and 10, respectively (Supplementary Fig. 12). The calculated *E*_α_, *E*_R_, and *ƞ*_solar thermal_ of the fabricated SGM absorbers are close to those of ideal H2 SGM absorber with a 30-nm graphene metamaterial layer [*E*_α_/*E*_R_(*C* = 1)/*ƞ*_solar thermal_(*C* = 1)/*ƞ*_solar thermal_(*C* = 10) = 90.9%/3.8%/86.9%/90.5%] (Supplementary Table 4), which are calculated based on the simulated absorbance spectra, at the same working situations. The solar-to-thermal efficiencies of the SGM absorbers can remain stable (with <2% variation) at working temperature of 100 °C in air over prolonged period. The thermal stability of SGM absorbers can be further proved by the Raman scattering and X-ray diffraction (XRD) techniques. As shown in Fig. 5e, over 24 h of heating in air at 100 °C, the Raman spectra of SGM absorbers with and without thermal treatments keep almost the same. Moreover, no Raman peak and XRD pattern of copper oxide^38–41^ in the short wavenumber regime of Raman spectra (Fig. 5e) and XRD spectra (Supplementary Fig. 13) were observed, suggesting that the graphene metamaterial layer could successfully protect the underlying Cu substrates to avoid oxidation at high temperatures in air.

Supplementary Table 5 compares the absorbance and thermal characteristics and structural factors of 3D SGM absorber with those of the state-of-the-art absorbers^10,11,18,19,29^. SGM absorber has excellent structural continuity, solar-selective absorption property, and thermal conduction capability, combined with sufficient solar absorption and suitability for large-scale fabrication. Therefore, the herein demonstrated 3D SGM absorbers have promising potentials for applications in the fields related to solar-thermal energy harvesting.

## Discussion

We have proposed, modeled, and experimentally demonstrated the 3D SGM for solar-selective absorption. Realizing near-unity absorption across the UV and NIR spectral ranges and simultaneously minimizing the emissivity in the IR regime allow significant improvement of solar energy collection efficiency and reducing the thermal radiation loss by the SGM absorbers. High solar-to-thermal conversion efficiency of 90.1%, overall efficiency of 68.9%, water evaporation rate of 1.5 kg m^−2^ h^−1^, and solar-to-vapor efficiency of 96.2% have been achieved, respectively, which represent the state-of-the-art performance. Therefore, the SGM strategy simultaneously tackles the major challenges of efficient solar-thermal absorber and enables omnidirectional absorption, flexible tunability of wavelength-selective absorption, and achieves excellent photothermal performance and thermal stability. In addition, as the structural parameters are the main determining factors of the overall absorption performance of the SGM absorber instead of the intrinsic properties of the metal substrate, the design gives the flexibility in choosing different metal substrates according to application needs. Although we demonstrated the use of copper substrate in this study, aluminum foil can also be used for the purpose of cost saving by slightly optimizing the structural parameters. Furthermore, the structural parameters can be readily tuned coping with different applications, such as thermal shield and specialized energy absorbing materials, according to the design principle. Therefore, these attractive properties of SGM absorbers suggest promising broad applicability in many fields related to energy harvesting and manipulation.

## Methods

### Sample preparation

The proposed solar-thermal absorbers were fabricated on the Cu substrates. At first, the patterning process was performed using conventional laser nanofabrication technology^32^. A femtosecond laser beam (Libra, 100 fs pulse, 10 kHz repetition rate, 800 nm wavelength) was focused by a high numerical aperture (NA) objective lens (×100, NA = 0.85) onto the Cu substrate, which was mounted on a 3D nanometric piezo-electric stage (Physik Instrumente®), to fabricate trench-like structures. The conditions for the laser nanofabrication process were laser power of 70 µW (i.e., a pulse energy of 7 nJ); scanning speed of 30 µm s^−1^; and scanning direction for each trench of −*Z*-direction (bottom-up). After patterning, the structured Cu substrate was cleaned using DI water to remove any residual particles. Next, a 40-nm film of GO was deposited by SA GO coating process^30^. In this process, the sample was sequentially placed in aqueous poly(diallyldimethylammonium chloride) (PDDA) solution, DI water, GO solution, and DI water. A layer of aqueous PDDA with positive charge is required to deposit on the Cu substrate first, to allow the firm attachment of the GO flakes and, thereby, ensure good contact between the Cu and GO layer. By repeating this process, multi-layer GO film was deposited on the structured Cu substrate. The conditions for the SA GO coating process are described as follows: the immersion time in each step: 30 s; the repeated times of the process: 10; the concentrations of PDDA and GO solutions: 2% and 3 mg mL^−1^, respectively. Finally, the GO-coated sample was illuminated with a mercury lamp (Olympus U-RFL-T) for 2 h to reduce the GO film, providing a solar-thermal absorber possessing 3D SGM with a 30-nm graphene metamaterial layer.

### Sample characterization

Top-view profiles of the SGM samples were observed using a field emission SEM (RAITH150 Two). The spectral reflectance and transmittance of SGM samples were measured using a UV–visible–NIR spectrophotometer (PerkinElmer Lambda 1050 UV/VIS/NIR Spectrometer) with an integrating sphere and an FTIR spectrometer (Bruker Hyperion 2000). The absorbance (*A*) was calculated from the measured reflectance (*R*) and transmittance (*T*) using the equation: *A* = 1 − *R* − *T*. The optical constants of graphene metamaterial were obtained by fitting the data measured from ellipsometer (J.A. Woollam M-2000) and FTIR spectrometer. SGM samples were heated in a furnace (Thermo Scientific Thermolyne Muffle Furnace) at a temperature of 100 °C in air for 12 and 24 h to test their thermal stability. The micro-Raman spectra of SGM samples before and after thermal annealing treatments were recorded using a spectrometer (NT-MDT Spectrum Instruments NTEGRA SPECTRA) with a 532 nm excitation line. XRD measurements were carried out by a diffractometer (Bruker D8 Discover) using the Cu-Kα line (*λ* = 0.154 nm) in the *θ*–2*θ* geometry.

### Numerical simulation

In simulations, we set the plan waves with the wavelengths from 0.28 to 25 µm propagating from 2 µm above the SGM to the SGM-coated Cu substrate, which is one of the most used substrates in solar-thermal power generation systems. To further study the absorption characteristics, we setup two detectors for each 3D SGM: the first, 1 µm below the graphene metamaterial−Cu interface (inside the Cu substrate); the second, 1 µm above the incident light (in the air). These two detectors recorded the transmittance (*T*) and reflectance (*R*) of the SGM, respectively, from which we could calculate the absorbance (*A*) by the equation *A* = 1 – *R* – *T*. In the simulations, we obtained the optical constants for Cu from the literature^42^ and for graphene metamaterials from the ellipsometry and FTIR measurements. For details, please see Supplementary Fig. 14. The absorption of the SGM absorber under the unpolarized light was calculated by averaging the absorptions of the absorber under illumination with TE- and TM-polarized light.

### Thermal measurements

Samples were illuminated with a solar simulator (Newport Sol 3A^TM^, power density: 1000 W m^−2^) in an open environment. The temperatures of the SGM absorber and flat GM@Cu foil were recorded using a thermal imaging camera (Testo 890) after various durations of time. In the experiment, we used the measured emissivity to calibrate the thermal imaging camera. After the calibration, the initial temperatures of each sample are the same (ca. 20.7 °C) before the sunlight illumination. The mass change of water under one sun illumination was recorded by an electronic balance (OHAUS Pioneer® Analytical).

## Supplementary information

Supplementary Information

## Data Availability

All relevant data are available from the corresponding author upon reasonable request.

## Code availability

The code used to calculate the results for this work is available from the corresponding author upon reasonable request.
