# Supplementary material for: Structured graphene metamaterial selective absorbers for high efficiency and omnidirectional solar thermal energy conversion
Source: Nat Commun. 2020 Mar 13;11:1389. doi: 10.1038/s41467-020-15116-z (PMC7069956; doi:10.1038/s41467-020-15116-z)
Supplement: Supplementary file 1 — Supplementary Information [file 41467_2020_15116_MOESM1_ESM.pdf]

Supplementary Information

Structured graphene metamaterial selective absorbers for high efficiency and omnidirectional solar thermal energy conversion

Lin et al.

Supplementary Note 1: Correlation between CO_2 emission and efficiency of various power plants

Traditional power plants, which generate energy by burning fuel (e.g. coal, natural gas, and oil) to release heat, have high efficiencies (about 40% to 50%)[1]. However, these electricity generation technologies are not environmentally friendly since their high carbon dioxide (CO_2) emissions (red-color region). In recent decades, the technologies of renewable energy, such as solar photovoltaic and wind energy, are widely used and investigated because of their low CO_2 emissions, but the limitation of these technologies are the low efficiencies (blue-color region)[1]. Based on the previous technologies, solar thermal energy technology overcomes the disadvantages of previous methods, as displayed in Fig. S1, it features high efficiencies of more than 60%, while low CO_2 emissions (green-color region). Therefore, the investigation and applications of solar thermal energy technology become more and more important in recent years.

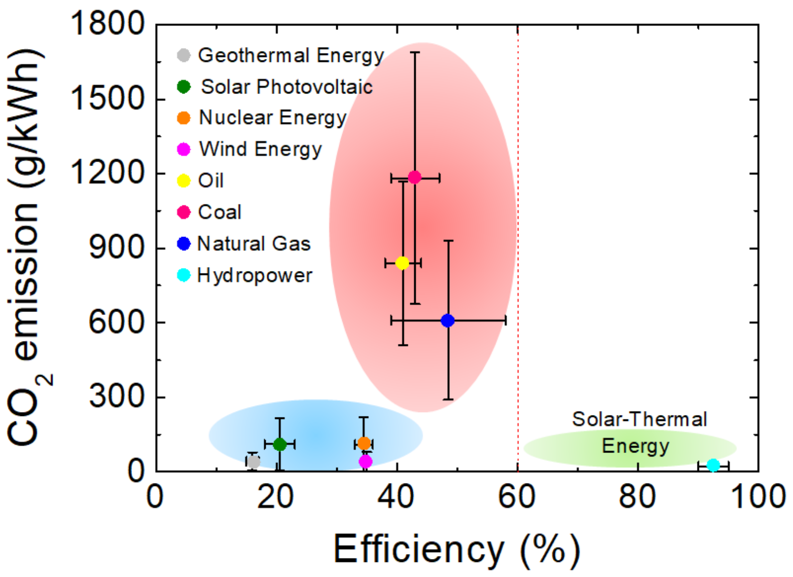

Supplementary Figure 1. Correlation between CO_2 emission and efficiency of the power plants with distinct electricity generation technologies.

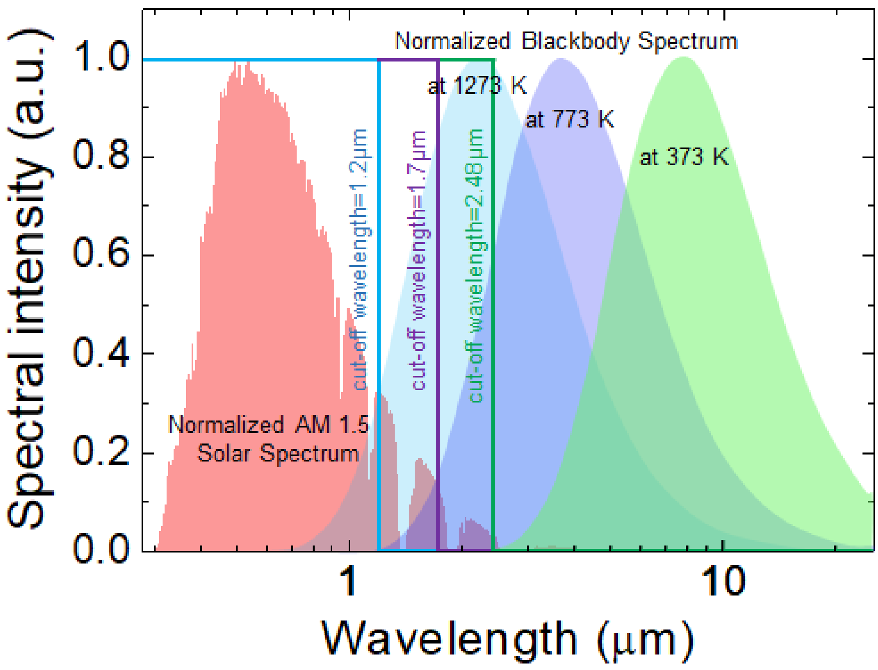

Supplementary Figure 2. Normalized spectral intensities of the AM 1.5 solar spectrum and the blackbody radiation spectra at 373, 773, and 1273 K. The cut-off wavelengths of the ideal solar absorber at a working temperature of 373, 773, and 1273 K are 2.48, 1.7, and 1.22 μm, respectively.

Supplementary Note 2: Strategies of solar selective absorbers: multi-layer thin film coating, plasmonic nanostructures, and carbon-based materials.

In general, solar selective absorbers can be divided into three distinct types based on their thermal modulation strategies: multi-layer thin film coating[2-4], plasmonic nanostructures[5-9], and carbon-based materials[10-15]. Multi-layer thin-film coating-type strategy is commonly employed to achieve solar selective absorption for solar-thermal energy conversion. This type of strategy usually uses the physical vapor deposition (PVD) system to deposit the multi-layer thin-film materials, e.g. chromium (III) oxide (Cr_2O_3), aluminum oxide (Al_2O_3), silicon oxide (SiO_2)[2-4], on the substrate. Although good spectral selectivity can be achieved with high absorptions in nanometric device architectures, the nature of the non-scalable, low cost-effective, and low tolerance vacuum fabrication method makes this strategy less attractive for practical solar-thermal conversion and thermal management.

The development of plasmonic structures provides a promising approach for photothermal modulation technology[5-9,16-20]. Many metamaterial-based structures are being extensively investigated and have demonstrated tailorable high absorbance over broad bandwidth[16,17,19]. In general, metallic metamaterial-based resonators are three-layered structures that create a resonant cavity in the structure. The three-layered structures comprise sophisticated isolated metal nanostructures on a dielectric layer/ flat metal film stack for fine-tuning of the electromagnetic (EM) properties of such structures. These isolated metal structures result in undesired high absorption of the photo-generated thermal energy trapped locally by the discontinuous metallic structures, which could not be transported to other sites for effective conversion or dissipation. In addition, metamaterials operation mechanism requires the dimensions of the metallic structures to be much smaller than the EM absorbance wavelengths in the visible to

near-infrared (NIR) region, which limits the feature sizes to be ~ 10 to 100 nm[16,17,20], producing stringent requirements for fabrication process based on focused ion beam (FIB) or electron beam lithography (EBL)[17-20]- both of which are not scalable and of high cost. Therefore, the metallic metamaterial approach is less practical for real-life thermal management.

Recently, low-cost and scalable EM absorbers based on chemically synthesized adsorbing metallic/dielectric nano-particles (NPs), such as gold (Au) and aluminum (Al), onto a porous membrane or polymer matrix[21,22] have been demonstrated. These NPs-stacked absorbers exhibited high and broadband absorbance from visible to NIR regime (wavelength from 0.4 to 2.5 μm). Nevertheless, the tunability and selectivity of the absorption bands of these structures are limited by localized surface plasmon resonance (LSPR) and the porous supporting membrane. Furthermore, the thermal transport inside the volumetric stacks is not effective for heat conversion due to the in accessible surface areas. Therefore, the performance of such absorbers is significantly limited.

In the past few years, carbon-based absorbers with low-cost and scalability, have been demonstrated with high absorption over ultra-broadband wavelength regime[10-15]. These graphene-based absorbers featuring high absorption over broad wavelength regime means they do not have spectral selective mechanism and face the high thermal emission loss. Furthermore, the large thickness prevents the effective transport of the photothermal energy generated on the surface. Accordingly, the overall photo-generated heat could not be efficiently used and stored. Besides, the optical behaviors of the thick carbon-based absorbers would hardly be manipulated by the plasmonic structures due to the effective zone of near-field phenomena of metallic nanostructures, which only limits to several tens to hundreds nanometres.

Supplementary Note 3: Thermal conductivities of various metals and graphene

How to efficiently exploit the photogenerated heat is also one of the important factors in the design of solar-thermal absorber. In general, the graphene-based materials exhibit much better thermal conductivity than other metals, including Au, silver (Ag), Al, copper (Cu), and nickel (Ni). As displayed in Table S1, the thermal conductivities of suspended graphene and graphene on substrate are approximately 9.3 to 48.3 times and 1.4 to 7.2 times, respectively, larger than those of other metals[23-25]. In addition, we based on the densities and specific heats of metals and graphene-based materials to calculate the temperature increments in the metal films with respect to that in the graphene-based materials under consideration of the same volume and at the same thermal energy. Compared with the other metals, the graphene provides the highest temperature increments, which are 1.5 to 2.5 times higher than those of metals. Therefore, the graphene-based materials feature not only high temperature increments under illumination but also active function to efficiently transport the photo-generated heat due to the excellent thermal conductivity.

Supplementary Table 1. Thermal conductivities, densities and specific heats of various metals and graphene; temperature increments in those metal films with respect to that in graphene film under considerations of the same volume and at the same thermal energy.

**Material**	**Thermal conductivity ($W\ m^{-1}\ K^{-1}$)**	**Density ($g\ cm^{-3}$)**	**Specific heat ($J\ kg^{-1}\ K^{-1}$)**	$T_{Metal}/T_{Graphene}$
**Al**	247	2.7	897	0.6503
**Au**	317.9	19.3	129	0.6326
**Ag**	428	10.5	235	0.6383
**Zn**	113	7.1	338	0.5685
**Ni**	82.9	8.9	444	0.3986
**Cu**	401	8.96	385	0.4566
**Graphene**	2000-4000 (suspend)<br>~600 (on substrate)	2.25	~700	1

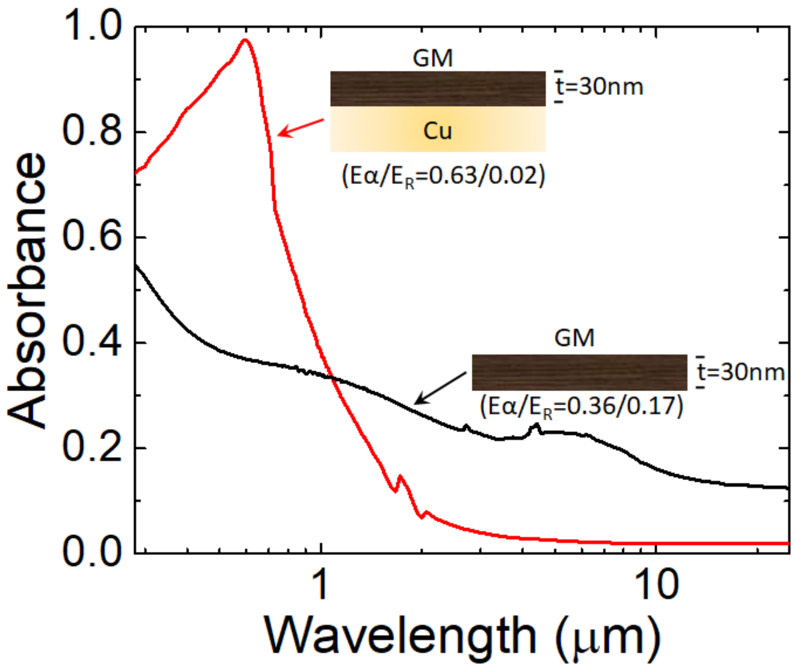

Supplementary Figure 3. Simulated absorbance spectra of free-standing 30-nm graphene metamaterial (GM) film and 30-nm GM film coated on Cu substrate based on the measured refractive index *n* and extinction coefficient *k* values. Total solar absorbances (E_α) and thermal radiation loss (E_R) of free-standing 30-nm GM film and those of 30-nm GM film coated on Cu substrate are calculated based on simulated absorbance spectra (Fig. S3), at working situations of T_A=373 K and C=1.

Supplementary Table 2. The labels of the SGM absorbers in terms of distinct dimensions.

**Symbol**	**H1**	**H2**	**H3**
	H045P06D08	H05908D1	H079P1D2
**Hole width (μm)**	0.45	0.59	0.79
**Period (μm)**	0.6	0.8	1
**Depth (μm)**	0.8	1	2

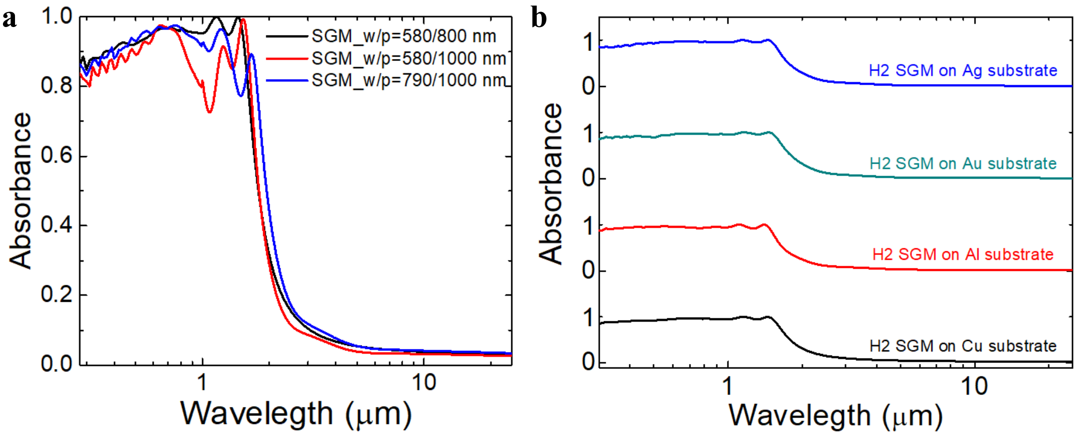

Supplementary Figure 4. (a) Simulated absorbance spectra of the SGM absorbers featuring various dimensions of *w* and *p*, while the trenches depth (*d*=1000 nm) and thickness of GM film (*t*=30 nm) were kept constant. The cut-off wavelengths of the 3D SGM absorber are mainly determined by the width of SGM. Furthermore, the larger value of $p - w$ would lead to lower absorbances in the UV to NIR regime. (b) When the thickness of GM layer is 30 nm, the optical behavior of the SGM is barely affected by the nature of the substrate [e.g., Au, Ag, Al, and Cu], suggesting that the substrate could be selected with a large degree of freedom.

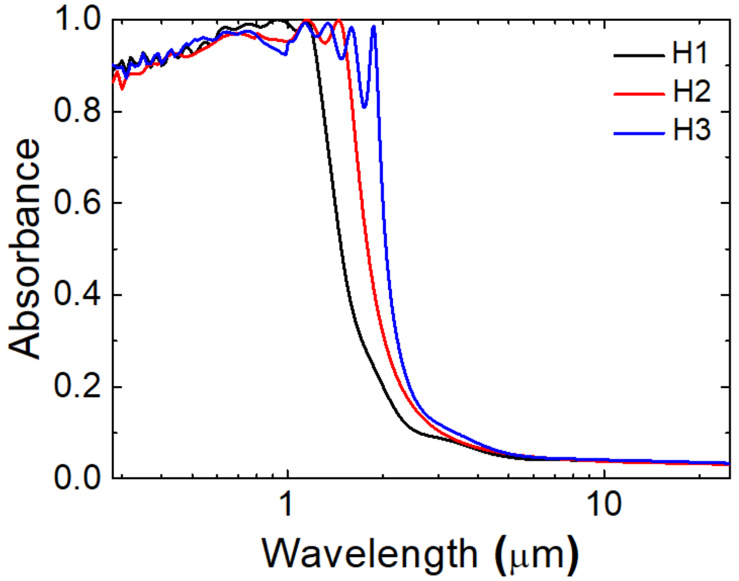

Supplementary Figure 5. Simulated absorbance spectra of the SGM absorbers featuring various dimensions of *w*, *p*, and *d*, while the thickness of GM film was kept constant (*t*=30 nm).

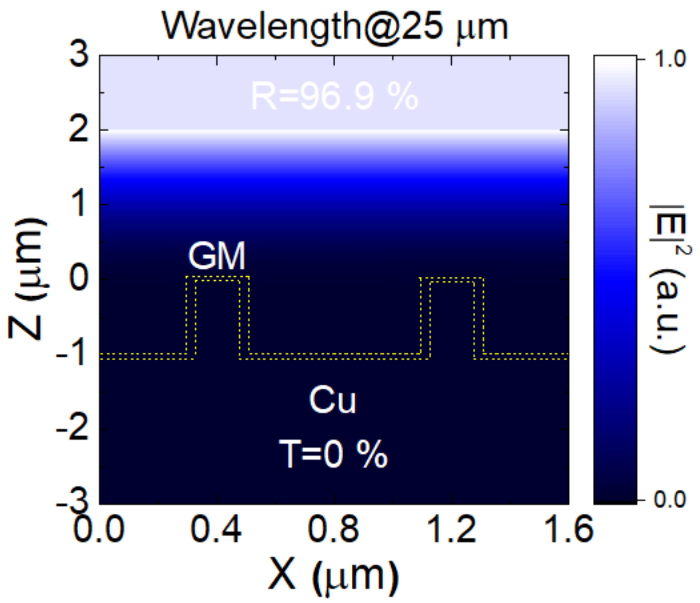

Supplementary Figure 6. The electric field distribution of H2 SGM absorber, which having thickness of GM layer of 30 nm, at a wavelength of 25 μm.

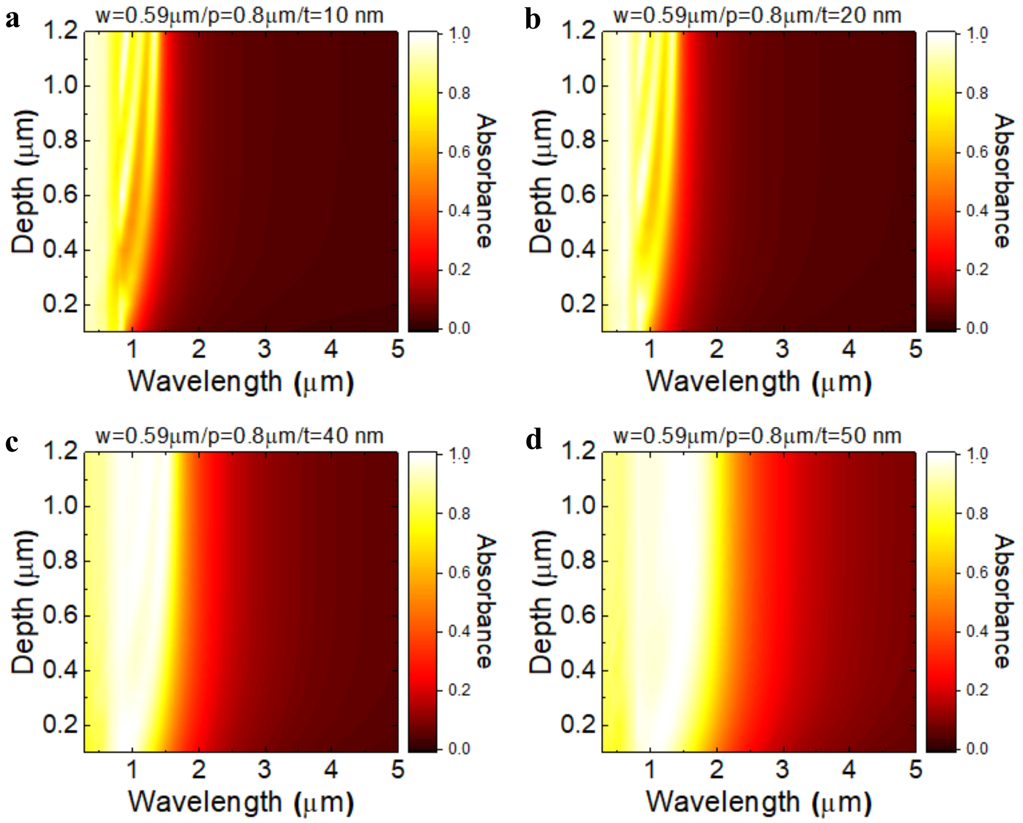

Supplementary Figure 7. Absorption spectra for SGM absorbers featuring hole width of 0.59 μm, period of 0.8 μm, and thickness of GM film of (a) 10 nm, (b) 20 nm, (c) 40 nm, and (d) 50 nm, respectively, as a function of depth of trenches and wavelength.

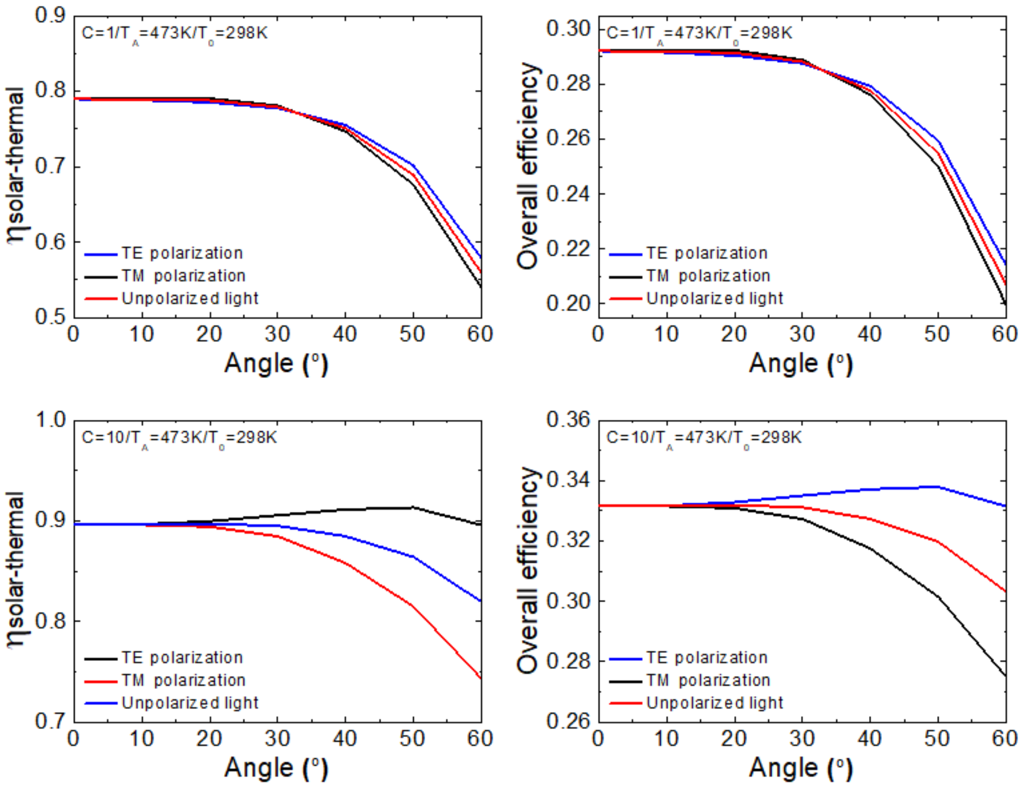

Supplementary Figure 8. The calculated solar-to-thermal conversion efficiencies and overall efficiencies of H2 SGM absorber with 30-nm GM layer under TE-polarized, TM-polarized, and unpolarized light at various angles of incidence (from 0 to 60°) and working situations (T_0=298 K, T_A=473 K, and C=1 and 10).

a b

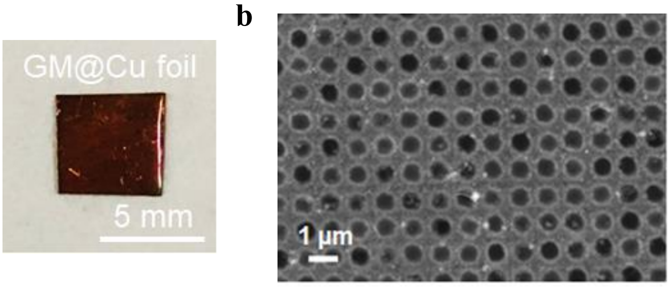

Supplementary Figure 9. (a) Photograph of Cu foil coated with a GM layer (GM@Cu foil). (b) Top-view SEM image of the SGM absorber featuring hole width of 0.6 μm and period of 0.8 μm.

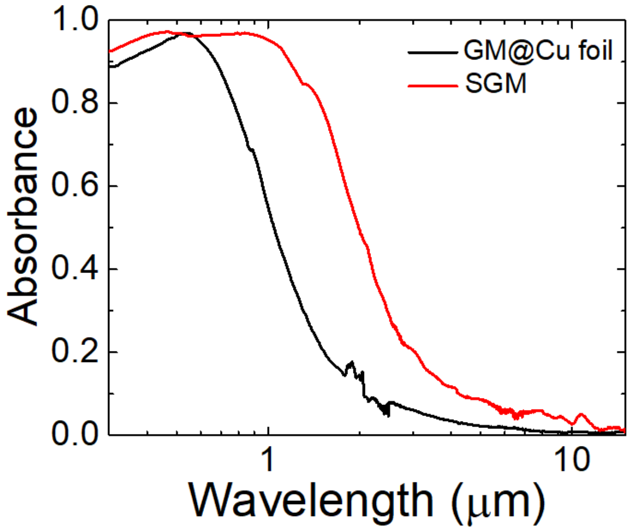

Supplementary Figure 10. Measured absorbance spectra of Cu foil coated with 30-nm GM layer and SGM absorber having 0.6-μm hole width and 0.8-μm period. When the Cu foil coated with 30-nm GM layer, the sample exhibits high absorbance in visible regime. However, the absorptions of Cu foil coated with 30-nm GM layer decrease dramatically in the NIR regime and are lower than 3% in the IR regime. In contrast, the SGM achieves solar selective absorption and performs high absorbances over broadband wavelengths, including UV, visible, and NIR regime, while keep low absorbances in the IR regime. Therefore, the SGM can efficiently collect sunlight and sufficiently avoid energy loss from thermal radiation.

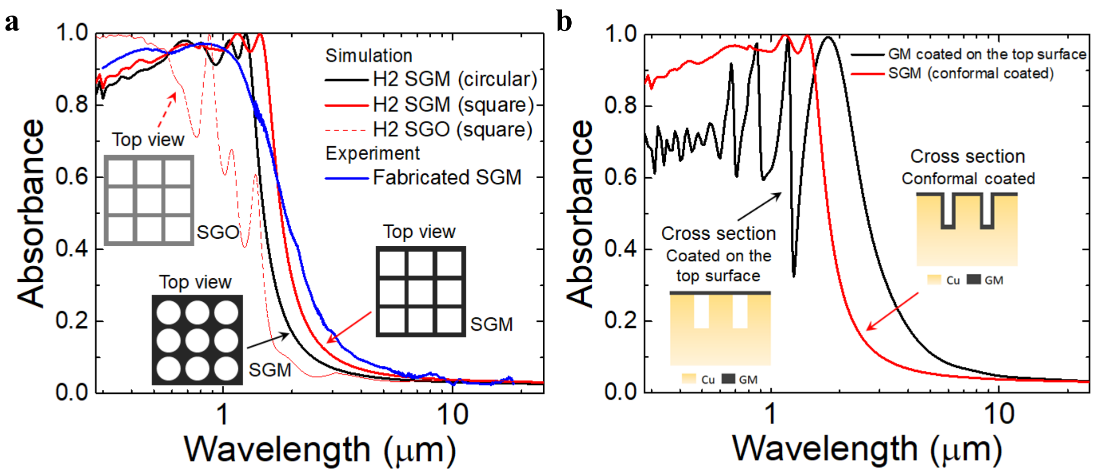

Supplementary Figure 11. (a) Simulated spectral absorbance for the H2 SGM absorber with 30-nm GM film featuring circular holes (black line) and square holes (red line), and the H2 structured graphene oxide (SGO) with 30-nm graphene oxide (GO) film featuring square holes (red dash line). The measured absorbance spectra of the fabricated SGM absorber is slightly different from the simulated results, presumably because the roughness of the sidewalls and the shapes of holes and trenches in the fabricated SGM absorber differ from those in the simulations. (b) Simulated absorbance spectra of the trench-like metallic structures having width of 650 nm, period of 800 nm, trench depth of 1 μm, and a 30-nm graphene metamaterial layer on the top surface (non-conformal, black line) and the H2 SGM absorber (red line). Both structures feature square holes.

Supplementary Table 3. Total solar absorbances (E_α), thermal radiation loss (E_R), and solar-to-thermal conversion efficiencies ($\eta_{solar\text{-}thermal}$) of SGM absorber before and after thermal stability tests at working situations of T_A=373 K, and C=1 and 10.

Time (hour)	Thermal stability test (at 100 ℃ in air)			
	**E_α**	**E_R (C=1)**	**$\eta_{solar\text{-}thermal}$ (C=1)**	**$\eta_{solar\text{-}thermal}$ (C=10)**
**0**	0.905	0.041	0.863	0.901
**12**	0.911	0.039	0.871	0.906
**24**	0.919	0.038	0.881	0.915

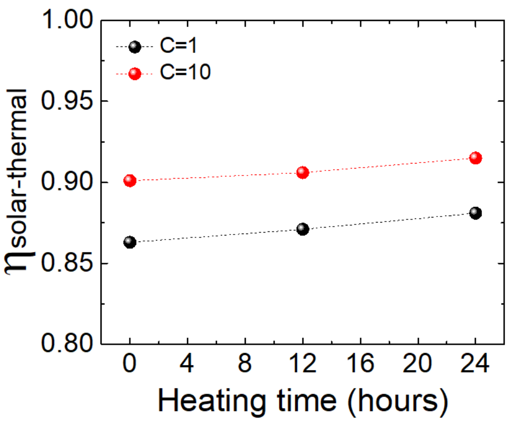

Supplementary Figure 12. Calculated solar-to-thermal efficiencies at working situations of T_A=373 K, and C=1 and 10 of fabricated SGM absorber before, after 12 hours, and after 24 hours of heating at 100 °C in air.

Supplementary Table 4. Total solar absorbances (E_α), thermal radiation loss (E_R), and solar-to-thermal conversion efficiencies ($\eta_{solar\text{-}thermal}$) of ideal H2 SGM absorber with 30-nm GM layer, which are calculated based on simulated absorbance spectrum (Fig. 1c), at working situations of T_A=373 K, and C=1 and 10.

**E_α**	**E_R (C=1)**	**$\eta_{solar\text{-}thermal}$ (C=1)**	**$\eta_{solar\text{-}thermal}$ (C=10)**
0.909	0.038	0.869	0.905

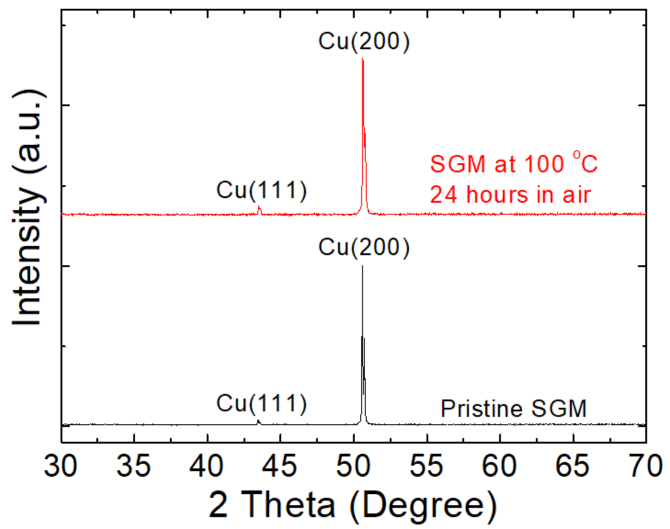

Supplementary Figure 13. XRD spectra of SGM absorbers before, after 12 hours, and after 24 hours of heating at 100 °C in air.

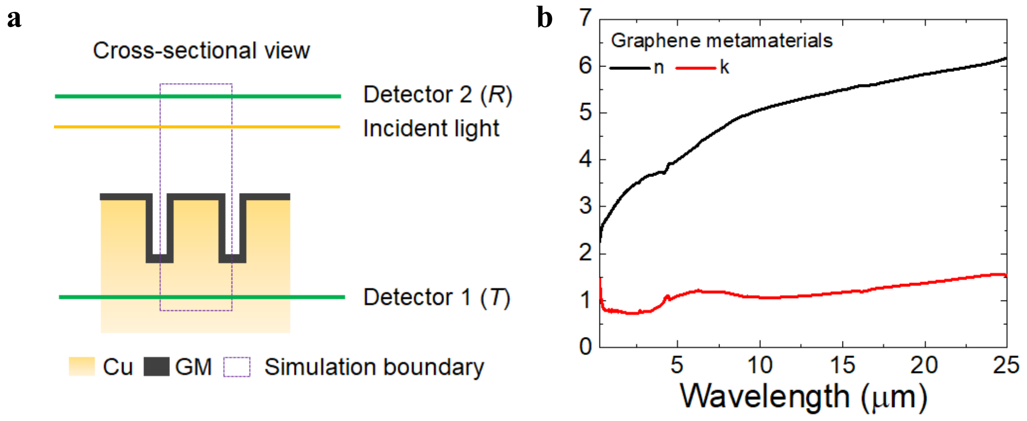

Supplementary Figure 14. (a) Schematic representation of the simulation setup. (b) Optical constants of graphene metamaterials used in the simulations, which are measured experimentally.

Supplementary Table 5. Performance and structural factors of state-of-the-art absorbers.

**Reference**	[16, 17]	[21, 22]	[26]	This work
**Structure type**	Metallic metamaterial	Nanoparticle-stacked absorber	Graphene grating metamaterial	3D SGM absorber
**Photothermal modulation strategies**	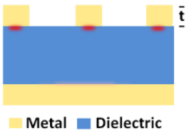	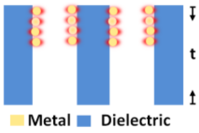	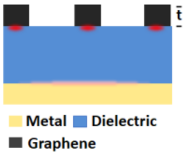	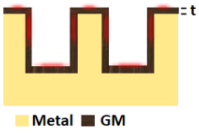
**Material**	Ag/polymer/Au [16]<br>Ag/SiO_2/Ag [17]	Al [21] or Au [22] /aluminium oxide	Graphene/SiO_2/Ag	Graphene metamaterial/Cu
**Schematic of spectrum**	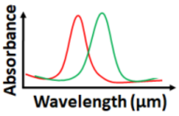	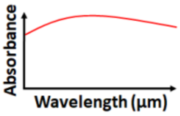	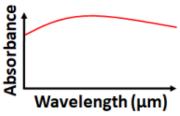	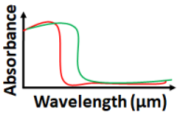
**Selective absorption**	Yes	No	No	Yes
**Bandwidth (A>50%)**	Narrow<br>(ca. 45 nm [16],<br>ca. 300 nm [17])	Broad<br>(>2 μm [21],<br>>2.5 μm [22])	Broad<br>(>2.5 μm)	Broad (tunable)<br>(H2: 1.51 μm,<br>H3: 1.77 μm)
**Thickness (t)**	ca. 70-100 nm	>10 μm	90 nm	30 nm
**Thermal conduction continuity**	No	No	No	Yes
**Scalable**	No	Yes	Yes	Yes
**Temperature under sunlight illumination**	N/A	N/A	ca. 160 °C	ca. 80 °C
**Solar-to-vapor efficiency under 1 sun illumination**	N/A	ca. 58% [21]<br>ca. 50% [22]	N/A	96.2%

Photo-generated heat

Supplementary References

1. Moomaw, W. et al. Annex II: Methodology. *IPCC Special Report on Renewable Energy Sources and Climate Change Mitigation* (2011).

2. Cao, F., McEnaney, K., Chen, G. & Ren, Z. A review of cermet-based spectrally selective solar absorbers. *Energy Environ. Sci.* **7**, 1615–1627 (2014).

3. Barshilia, H. C., Selvakumar, N., Vignesh, G., Rajam, K. S. & Biswas, A. Optical properties and thermal stability of pulsed-sputter-deposited Al_xO_y/Al/Al_xO_y multilayer absorber coatings. *Sol. Energy Mater. Sol. Cells* **93**, 315–323 (2009).

4. Selvakumar, N., Manikandanath, N. T., Biswas, A. & Barshilia, H. C. Design and fabrication of highly thermally stable HfMoN/HfON/Al_2O_3 tandem absorber for solar thermal power generation applications. *Sol. Energy Mater. Sol. Cells* **98**, 1–23 (2012).

5. Mandal, J. et al. Scalable, "dip-and-dry" fabrication of a wide-angle plasmonic selective absorber for high-efficiency solar–thermal energy conversion. *Adv. Mater.* **29**, 1702156 (2017).

6. Lin, K. T. et al. Loading effect–induced broadband perfect absorber based on single-layer structured metal film. *Nano Energy* **37**, 61–73 (2017).

7. Tsao, S. H. et al. White-light-induced collective heating of gold nanocomposite/bombyx mori silk thin films with ultrahigh broadband absorbance. *ACS Nano* **9**, 12045–12059 (2015).

8. Zhou, L. et al. 3D self-assembly of aluminium nanoparticles for plasmon-enhanced solar desalination. *Nat. Photonics* **10**, 393–398 (2016).

9. Zhou, L. et al. Self-assembled spectrum selective plasmonic absorbers with tunable bandwidth for solar energy conversion. *Nano Energy* **32**, 195–200 (2017).

10. Xu, N. et al. Mushrooms as efficient solar steam-generation devices. *Advanced Materials* **29**, 1606762 (2017).

11. Ghasemi, H. et al. Solar steam generation by heat localization. *Nat. Commun.* **5**, 4449 (2014).

12. Li, X. et al. Graphene oxide-based efficient and scalable solar desalination under one sun with a confined 2D water path. *Proc. Natl. Acad. Sci. U. S. A.* **113**, 13953–13958 (2016).

13. Hu, X. et al. Tailoring graphene oxide-based aerogels for efficient solar steam generation under one sun. *Adv. Mater.* **29**, 1604031 (2017).

14. Yang, Y. et al. Graphene-based standalone solar energy converter for water desalination and purification. *ACS Nano* **12**, 829–835 (2018).

15. Ren, H. et al. Hierarchical graphene foam for efficient omnidirectional solar–thermal energy conversion. *Adv. Mater.* **29**, 1702590 (2017).

16. Moreau, A. et al. Controlled-reflectance surfaces with film-coupled colloidal nanoantennas. *Nature* **492**, 86–89 (2012).

17. Aydin, K., Ferry, V. E., Briggs, R. M. & Atwater, H. A. Broadband polarization-independent resonant light absorption using ultrathin plasmonic super absorbers. *Nat. Commun.* **2**, 517 (2011).

18. Liu, N., Mesch, M., Weiss, T., Hentschel, M, & Giessen, H. Infrared perfect absorber and its application as plasmonic sensor. *Nano Lett.* **10**, 2342–2348 (2010).

19. Bossard, J. A. et al. Near-ideal optical metamaterial absorbers with super-octave bandwidth. *ACS Nano* **8**, 1517–1524 (2014).

20. Jiang, Z. H. et al. Broadband and wide field-of-view plasmonic metasurface-

enabled waveplates. *Sci. Rep.* **4**, 7511 (2014).

21. Zhou, L. et al. 3D self-assembly of aluminium nanoparticles for plasmon-enhanced solar desalination. *Nat. Photonics* **10**, 393–398 (2016).

22. Zhou, L. et al. Self-assembled spectrum selective plasmonic absorbers with tunable bandwidth for solar energy conversion. *Nano Energy* **32**, 195–200 (2017).

23. Gale, W. F. & Totemeoer, T. C. Smithells Metals Reference Book, *Elsevier Ltd* (2004).

24. Pop, E., Varshney, V. & Roy, A. K. Thermal properties of graphene: Fundamentals and applications. *MRS Bull.* **37**, 1275–1281 (2012).

25. Li, Q. Y. et al. Measurement of specific heat and thermal conductivity of supported and suspended graphene by a comprehensive Raman optothermal method. *Nanoscale* **9**, 10784–10793 (2017).

26. Lin, H. et al. A 90-nm-thick graphene metamaterial for strong and extremely broadband absorption of unpolarized light. *Nat. Photonics* **13**, 270–276 (2019).
